# A BRILLIANT-BRCA study: residual breast tissue after mastectomy and reconstruction

**DOI:** 10.1007/s10549-024-07425-4

**Published:** 2024-07-09

**Authors:** Orit Kaidar-Person, Renata Faermann, Dor Polikar, Kfir Cohen, Rinat Bernstein-Molho, Monica Morrow, Liesbeth Jorinne Boersma, Birgitte Vrou Offersen, Philip Poortmans, Miri Sklair-Levy, Debbie Anaby

**Affiliations:** 1 School of Medicine, Faculty of Medical & Health Sciences, Tel-Aviv University, Tel-Aviv, Israel; 2 Breast Radiation Unit, The Jusidman Cancer Center, Chaim Sheba Medical Center, Tel-Hashomer, Ramat-Gan, Israel; 3 GROW-School for Oncology and Reproduction, Maastricht University, Maastricht, The Netherlands; 4 The Merav High-Risk Clinic - Chaim Sheba Medical Center, Tel-Hashomer, Ramat-Gan, Israel; 5 Department of Diagnostic Imaging, Sheba Medical Center, Tel-Hashomer, Ramat-Gan, Israel; 6 Breast Cancer Institute, The Jusidman Cancer Center, Chaim Sheba Medical Center, Tel-Hashomer, Ramat-Gan, Israel; 7 Oncogenetics Unit, Institute of Genetics, Chaim Sheba Medical Center, Tel-Hashomer, Ramat-Gan, Israel; 8 Breast Service, Department of Surgery, Memorial Sloan Kettering Cancer Center, New York, NY USA; 9 Department of Radiation Oncology (Maastro), GROW Research Institute for Oncology and Reproduction, Maastricht University Medical Centre+, Maastricht, The Netherlands; 10 Department of Experimental Clinical Oncology, Aarhus University Hospital, Aarhus, Denmark; 11 Department of Radiation Oncology, Iridium Netwerk, Wilrijk-Antwerp, Belgium; 12 Faculty of Medicine and Health Sciences, University of Antwerp, Wilrijk-Antwerp, Belgium

**Keywords:** Mastectomy, MRI, Residual breast, Risk reducing mastectomy, Skin sparing, Nipple sparing, Breast tissue

## Abstract

**Introduction:**

Different types of mastectomies leave different amounts of residual breast tissue. The significance of the residual breast volume (RBV) is not clear. Therefore, we developed an MRI tool that allows to easily assess the RBV. In this study we evaluated factors associated with RBV after skin or nipple sparing mastectomy (SSM/NSM) in breast cancer *BRCA* pathogenic variant (PV) carriers who underwent both therapeutic and risk reducing SSM/NSM and its relation to breast cancer outcomes using an innovative MRI-based tool.

**Methods:**

Data of breast cancer *BRCA* PV who were treated between 2006 and 2020 were retrieved from of the oncogenetics unit databases. Only patients who underwent SSM/NSM and had a postoperative breast MRI available for analysis were included. Data collected included demographics, clinicopathological features, and outcomes. The MRI tool was developed by a breast cancer imaging laboratory. A logistic regression test and 95% confidence interval (CI) were used to assess the associated risk of increased RBV. A forward stepwise linear regression was used to correlate tumour-patient specific factors and RBV, and a Kaplan–Meier curve to show the probability of locoregional relapse.

**Results:**

A total of 84 patients undergoing 89 mastectomies were included. At a median follow-up of 98 months, 5 local, 2 regional, and 4 distant recurrences were observed. RBV was not significantly related with breast cancer outcomes (*p* value = NS). A higher body mass index (BMI) was associated with a higher RBV (*p* < 0.0001). A larger number of involved axillary nodes was associated with a smaller RBV (*p* = 0.025). The RBV on the risk-reducing mastectomy side was significantly higher compared to the breast cancer side (*p* value = 0.007). Local recurrences occurred in the vicinity of the primary tumour.

## Introduction

Residual breast tissue after mastectomy can be found in more than 50% of patients [1–4]. The extent of residual breast tissue is associated with breast density, breast size, body mass index (BMI), surgical expertise, type of mastectomy, surgical approach (e.g. more in case of inframammary incision), extent of axillary surgery, and method of evaluation (e.g. imaging type) [1–3, 5–7]. Evaluation of the amount and location of residual breast tissue after mastectomy have largely been done using imaging (e.g. breast magnetic resonance imaging, MRI) [8–13] and/or histologic examination [2, 4, 14–25]. Residual breast tissue has been identified after all types of mastectomies, and in all breast quadrants, regardless of preservation of the skin/nipple (skin or nipple sparing mastectomy, SSM/NSM) [3, 26, 27]. Nevertheless, SSM and NSM are associated with more residual breast tissue compared to total or modified radical mastectomy due to the nature of the surgical procedure [1, 2, 10, 28]. To preserve the breast skin in SSM, the surgeons need to separate the mammary gland from the subcutaneous fat at the level of the superficial fascia. This fascia is a very delicate and discontinuous structure that cannot usually be clearly identified at during surgery, so the plane is developed based on the estimated thickness of the subcutaneous fat layer [1, 2]. Underneath the nipple and areola complex (NAC), there is no fat layer, and the ducts extend from the glandular tissue into the nipple. Therefore, in case of NSM, to avoid harming the blood supply to the nipple, part of the glandular tissue might be preserved. The greater the amount of breast tissue left behind beneath the NAC the lower the risk of ischaemic necrosis, causing some surgeons to leave behind a substantial amount of breast tissue in order to minimize this risk [29]. Furthermore, the skin flap thickness may be also subjected to surgeon’s bias by aiming to achieve better aesthetic outcomes with a natural appearing neo-breast with thicker skin flaps [1, 2, 27].

The SKINI trial showed that surgical expertise, i.e. high-volume surgical practice, is important for obtaining a thin flap and desired aesthetic results without higher complication rates [1]. Nevertheless, residual breast tissue after mastectomy can reach a volume of 7.3% of the intact breast volume even in expert centres [1–3, 10]. Imaging studies show that the thicker the flap (skin and subcutis), the more residual *glandular* breast tissue remains behind, and some describe that the goal of removing all breast *glandular* tissue via SSM/NSM is an “unattainable goal” [7, 28]. The relationship of skin flap thickness to breast cancer free survival is not clear; however, a few reports suggest that non-radical surgery may be related to increased rates of breast cancer recurrence [30–32].

In our study on breast cancer patients who are *BRCA1* or *BRCA2* pathogenic variants (PV) carriers, we reported that the cumulative incidence of ipsilateral breast tumour recurrence (local recurrence) as *first* failure was more common in patients who underwent SSM/NSM and did not receive postoperative radiation therapy (RT). The rates of local recurrences in this SSM/NSM group without RT were significantly higher than generally reported in the literature and higher than those of *BRCA* PV carriers breast cancer patients who underwent breast conserving therapy (BCT) (15.3% after SSM/NSM without RT versus 5.2% after BCT, *p* = 0.049). None of the SSM/NSM group that were treated with postmastectomy RT had a local recurrence, even though this group had a higher tumour/nodal stage and the longest follow-up compared to SSM/NSM group [30].

The current Brilliant study was designed to develop a breast MRI-based artificial intelligence (AI) system that allows objective identification of residual breast tissue after mastectomy and implant-based breast reconstruction in large cohorts of patients. The aim of this BRILLIANT-BRCA pilot study was to apply this AI tool in our breast cancer *BRCA* PV cohort [30], in order to (1) evaluate the residual breast volume (skin flap volume) after SSM/NSM, (2) determine factors associated with a higher residual volume, and (3) assess if a higher flap volume was associated with ipsilateral locoregional relapses, or any other breast cancer event.

## Methods

All procedures performed in this study were in accordance with the ethical standards of the institutional research committee and with the 1964 Helsinki declaration and its later amendments or comparable ethical standards. Two ethics approvals were obtained by Sheba Medical Center ethics committee, one to develop the AI system which allowed us to process images, the second for retrieving images and patient information from the medical record. As this is a non-interventional study without direct patient contact, it was exempt from obtaining patients’ written informed consent.

### Development of AI-based segmentation of residual breast tissue

In short, programming was performed by AI programmer experts at the Breast Cancer Imaging Laboratory, department of diagnostic imaging at Sheba Medical Center (DP, KC, DA). An AI-based algorithm was developed to automatically segment residual breast tissue (fat and glandular tissue). Training was performed using anonymized non-fat suppressed T2-weighted breast MR images of intact breast, patients after breast augmentation, and after mastectomy and implant-based reconstruction. In the training set of MRIs after implant breast reconstruction, the residual breasts were manually segmented by two expert breast radiologists (RF, MSL), including repeated output reviews and corrections, to ensure accurate results of the AI-based segmentation to the “ground truth” (= breast radiologist). Subjective expert review and dice coefficient were used to assess the output of the system and correct the system repeatedly as needed. After the segmentation by the algorithm achieved a dice score of 94% agreement with an expert breast radiologist (validation process), and the breast radiologist approved correct segmentation, the system was used to evaluate the residual volume in the Brilliant BRCA study cohort. A full description of the AI process and system will be published upon completion of the project in compliance with recommendations for transparency in developing AI systems [33].

### Breast cancer BRCA PV patient cohort

A detailed description of the clinical data collection process has been published earlier [30]. Briefly, updated data of breast cancer *BRCA* PV carriers who were treated between 2006 and 2020 were retrieved from of the oncogenetics unit databases at Sheba Medical Center [30]. The MRIs were retrieved from Merav Center at Sheba, a multi-professional centre that includes breast imaging and high-risk clinics. Merav’s imaging protocol BRCA high risk after risk reducing mastectomy or breast cancer surgery includes a breast MRI 6 months after surgery alternating every 6 months with breast ultrasound and mammography [34].

Only patients who underwent implant-based reconstruction, for whom full data were available in the database, and a postoperative breast MRI was available at our institution for AI analysis, were included in this study. Recurrences were recorded as *first* event: local recurrences occurring synchronous with a distant event (i.e. < 3 months) or after a distant event were not recorded as local or regional recurrence but as distant event.

For evaluating the residual breast tissue volume, the first breast MRI done after mastectomy was used (~ up to one year from surgery) to reduce the possibility of atrophy of the residual tissue following therapy such as RT and effects of ovarian suppression.

### Residual breast volume measurement

The images used were retrospectively retrieved from a picture archiving and communication system (PACS, © Koninklijke Philips). All patient’s and imaging data were anonymized as follows: patient’s case report forms (CRF) and their images were given the same sequential “case number”. In case of bilateral breast cancer or contralateral risk-reducing mastectomy, each breast side was applied the same “case number” with a letter indicating the side of the breast (e.g. patient 12L, patient 12R). The residual breast volume analysis was done blindly to the clinicalpathological information and was blinded to whether or not it was a risk reducing mastectomy or a therapeutic mastectomy. Non-fat suppressed T2 MRI was used for the automatic segmentation on all axial levels and provided the residual breast tissue volume (cc/ml) (not including the implant).

### Statistical analysis

Descriptive statistics were used to describe study population, including for residual breast volume (percent, standard deviation, mean, median, etc.). *T*-test was used to evaluate the differences between patients’ characteristics (those with and without locoregional relapse), residual volume of right versus left breast cancer side, and the differences between the residual breast volume in risk-reducing mastectomy compared to the mastectomy breast cancer side with bilateral implant positioning. Logistic regression and 95% confidence interval (CI) were used to assess the associated risk of increased residual volume with relapse using an estimated Odds Ratio (OR) for an increase of 100 ml in volume. A forward stepwise linear regression was used to search for possible associations, and a correlation was calculated for tumour-patient specific factors. Covariates examined for explaining the residual breast volume included BMI, age at diagnosis, breast cancer diagnosis during pregnancy or lactation, diagnosis year, diagnosis via imaging versus palpation, tumour grade, tumour stage, nodal stage, receptor status (oestrogen receptor positive, HER2 positive, triple negative), axillary surgery, number of positive pathological nodes, number of removed nodes at surgery, chemotherapy, and RT. The dependent variable in the regression analysis was residual breast volume. Statistical significance was reported at the 5% level. Kaplan–Meier curve was used to show the probability of locoregional relapse per follow-up time for the whole cohort. Owing to low number of events, ipsilateral local and regional recurrences were evaluated together.

## Results

During the study period, of the 255 breast cancer *BRCA PV* mutation carriers, a total of 84 patients was eligible for the analysis. Overall, 89 breasts with breast cancers and implant-based reconstruction (5 synchronous cases), and 75 risk-reducing mastectomies with implant-based reconstruction were available. Of the 75 risk-reducing mastectomies, 44 (59%) were done at time of the breast cancer surgery, and the rest were performed within 12 months. Clinicopathological characteristics are shown in Table 1. All cases were reported to have pathologically confirmed tumour-free surgical margins.

**Table 1:** Clinicopathological characteristics of the whole cohort

*Age at diagnosis*	
Median (range)	38.4 years (28–63.5)
*Germline mutations*	*n* (%)
BRCA 1	58 (69%)
BRCA 2	24 (28.6%)
Both	1 (1.2%)
Unknown BRCA type	1 (1.2%)
*Breast cancer side*	*n* (%)
Left breast	43 (51.2%)
Right breast	36 (43%)
Bilateral	5 (5.8%)
*Histology*	*n* (%)
Pure DCIS	13 (15.5%)
IDC	59 (70.2%)
ILC	3 (3.6%)
DCIS + IDC	8 (9.5%)
IDC + ILC	1 (1.2%)
*Tumour grade of invasive cancer*	*n* (%)
Grade 1	**–**
Grade 2	12 (17%)
Grade 3	59 (83%)

*IDC* invasive ductal carcinoma, *ILC* invasive lobular carcinoma, *DCIS* ductal carcinoma in situ

At a median follow-up of 98 months (range 33–190 months), out of 89 mastectomies, 5 (5.6%) had local recurrences and 2 (2.2%) had regional recurrences. Of the 84 patients, 4 (4.7%) had a distant recurrence. Table 2 lists the clinicopathological characteristics of patients who had a locoregional relapse versus those who did not.

**Table 2:** Patient and tumour characteristics among those who had a locoregional relapse and those who did not

	Total		No locoregional recurrence		Locoregional recurrence		*p*
	*N*		*N*		*N*		
Median age at diagnosis in years (IQ range)	89	38.7 (34.2–44.6)	82	38.7 (34.5–44.9)	7	37.0 (28.9–40.7)	0.1476
BMI median (kg/m^2^) (IQ range)	81	23.6 (21.3–27.3)	75	23.6 (21.3–27.3)	6	23.2 (20.8–32.2)	0.9281
Residual breast volume (ml)^a^ median (IQ range)	89	368.4 (235.9–521.5)	82	373.4 (233.3–521.5)	7	348.6 (303.4–619.5)	0.5421
Diagnosis by Palpation *N* (%) Imaging *N* (%)	89	30 (33.7)	82	27 (32.9)	7	3 (42.9)	0.6370
	89	52 (58.4)	82	49 (59.8)	7	3 (42.9)	
Grade3 *N* (%)	89	63 (75.9)	77	59 (76.6)	6	4 (66.7)	0.5828
Triple-negative breast cancer *N* (%)	89	52 (59.8)	81	50 (61.7)	6	2 (33.3)	0.1711
Pregnant or lactation^b^ *N* (%)	89	11 (12.4)	82	10 (12.2)	7	1 (14.3)	0.8718
Chemotherapy *N* (%)	89	60 (67.4)	82	58 (70.7)	7	2 (28.6)	0.0223
Radiation therapy *N* (%)	89	22 (24.7)	82	22 (26.8)	7	0 -	0.1142

^a^Breast cancer side, 50% IQ interquartile range

^b^Pregnant or lactation—at time of breast cancer diagnosis

**Fig. 1:**
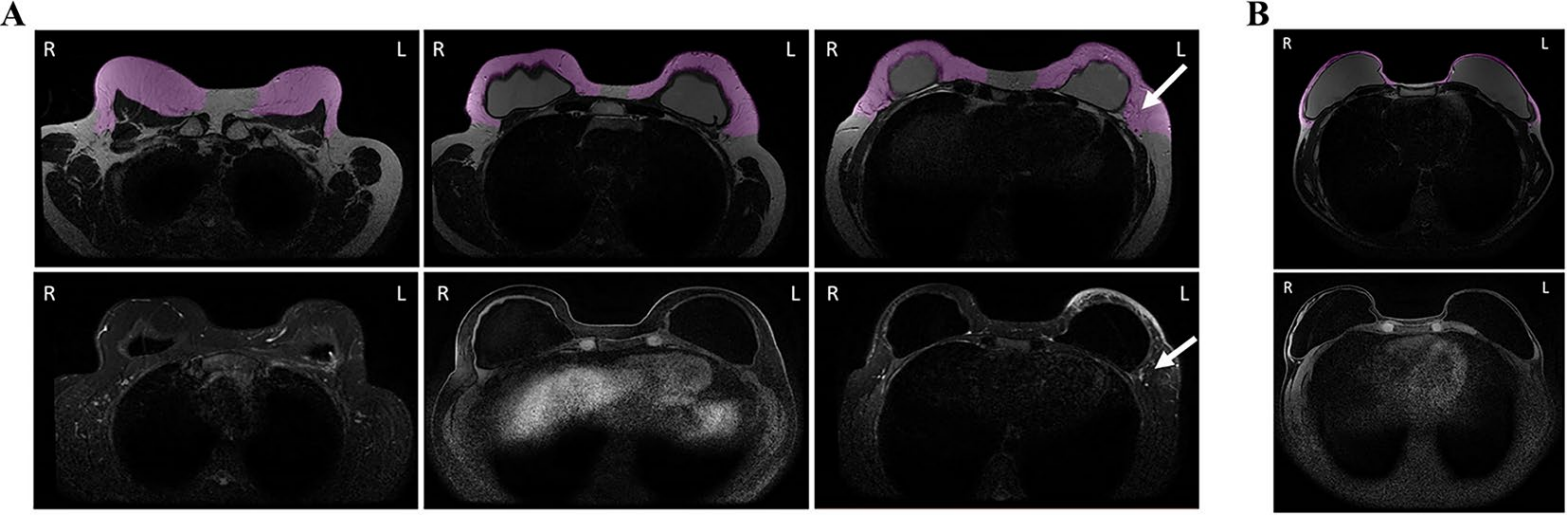
**A, B** The breast volume segmented by the Artificial Intelligence system (Colour wash in the upper panels, non-fat-suppressed T2 MRI) and the Breast MRI used for the segmentation (lower panels, T1 gadolinium MRI) are presented to illustrate the volumes of the flap of two cases. **A** A case of left-side breast cancer (residual breast tissue volume measured 742 ml), with right-side risk reducing mastectomy (residual breast tissue volume measured 708 ml). The white arrows show the perforating blood vessels that supply the breast tissue. **B** A case of left-side breast cancer (residual breast tissue volume measured 233 ml), right-side risk reducing mastectomy (residual breast tissue volume measured 290 ml)

The average residual breast volume measured by the AI system among those who had a locoregional relapse was 426.6 ml (SD = 154.8) versus 395.1 ml (SD = 197.1) in those without locoregional relapse (*p* value = 0.682). Figure 1 shows the algorithm segmentation in two patients, one with a thick flap (Fig. 1A) and one with a thin flap (Fig. 1B), also demonstrating that thickness varies between the breasts and at specific locations within the reconstructed breast.

Areas that tend to have most residual tissue were found to be located lateral, cranial, and caudal to the implant (Fig. 1). There were no significant differences in the residual breast volume for left (median: 372 ml) versus right therapeutic mastectomies (median: 368 ml) (*p* value = 0.74).

A logistic regression to assess the Odds Ratio (OR) for locoregional relapse (per breast side) per increase of 100 ml in volume resulted in an OR of 1.08 (95% CI 0.74–1.59; *p* value = 0.68). After censoring for RT and ductal carcinoma in situ (DCIS) because of no local recurrences in these groups (*n* = 54) from the logistic regression analysis, the estimated OR per 100 ml increase of volume was 1.03 (95% CI 0.68–1.56; *p* value = 0.89).

To analyse the association of residual breast volume with any breast cancer outcome (local, regional, and distant), women who had cancer on both sides were included once. There was a total of 12 breast cancer events (14.3%) in 84 patients during the follow-up period. The average volume among those who had any breast cancer event was 415.4 ml (SD = 193.8) versus 401.7 ml (SD = 198.0) in those without any breast event (*p* value = 0.78). The estimated OR for an increase of 100 ml in volume was 1.05 (95% CI 0.78–1.43; *p* value = 0.73) for any breast cancer event.

**Fig. 2:**
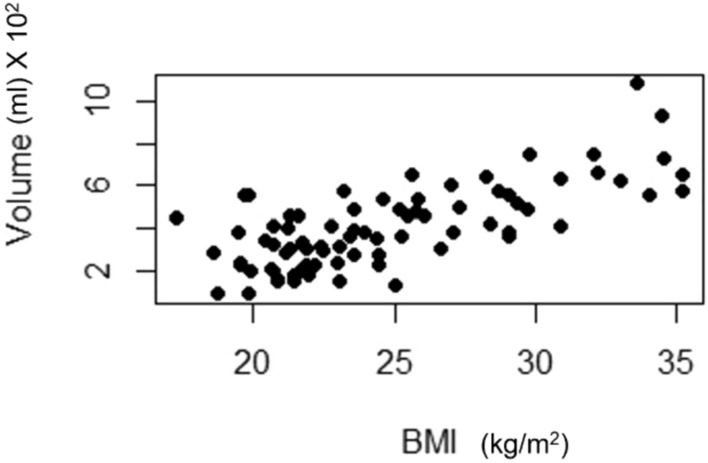
The correlation between body mass index (BMI) and residual tissue volume (per 100 ml)

A forward stepwise linear regression was used to assess clinicopathological factors possibly affecting the residual volume. None of the covariates listed above were found relevant, except BMI and the number of involved axillary nodes found at surgery. A higher BMI was associated with a *higher* residual breast tissue volume (*p* < 0.0001), and a larger number of involved axillary nodes was associated with a *smaller* residual breast tissue volume (*p* = 0.025). The model performance was strong for both (*R*^2^ = 0.51). Figure 2 plots the relation between BMI and residual breast volume, showing a strong correlation between residual breast volume and high BMI (kg/m^2^).

Comparison of the residual breast volume in risk-reducing mastectomy (median 410 ml, range: 106–1023 ml) versus therapeutic mastectomy (median 386.4 ml, range: 90–1080 ml) showed that the risk-reducing mastectomy had a significant higher volume compared to the breast cancer side (*p* value = 0.007). The difference in volumes had a reasonable symmetric distribution, and these results were similar when using a non-parametric test.

**Fig. 3:**
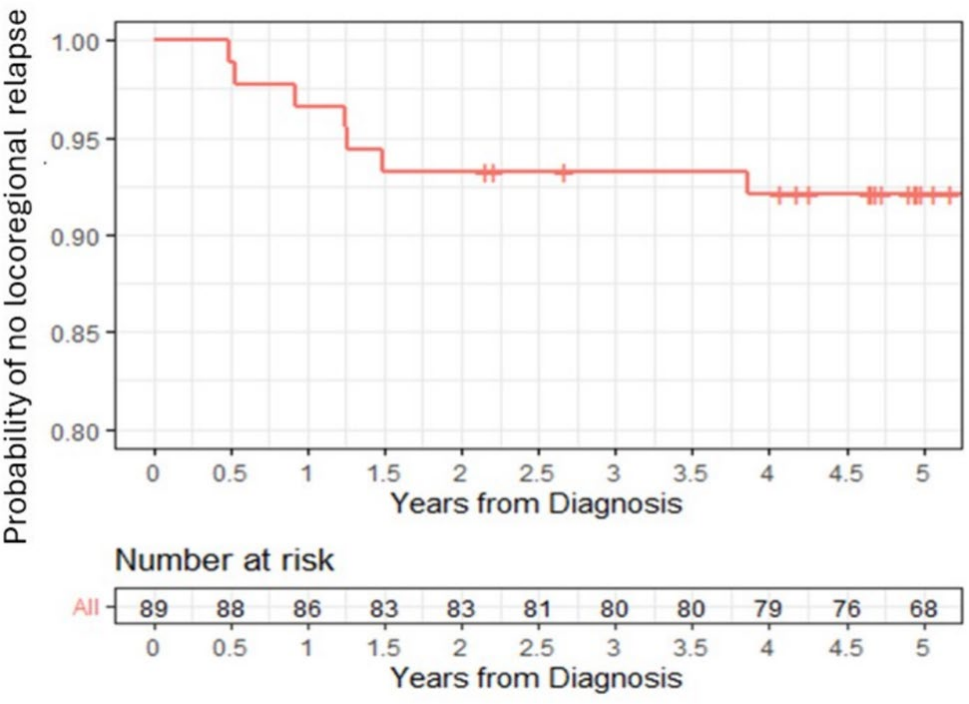
Kaplan–Meier curve showing the probability of locoregional-free survival per follow-up time

The time to locoregional recurrence is shown in Fig. 3, indicating that the locoregional relapses occurred early after surgery (most within 1.5 years). Of the 5 local recurrences, 3 cases had both the pre-therapy diagnostic images and images after local recurrence available for review. In all 3 cases, the recurrence occurred early, at the same breast quadrant/axial plane as the primary tumour.

**Fig. 4:**
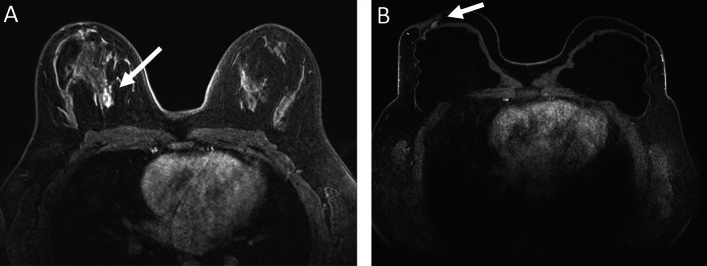
T1 gadolinium injected fat suppressed non-subtracted **A** MRI done at diagnosis, showing a tumour at upper central right breast, **B** MRI done at time of local recurrence, showing a tumour at the upper part of the right reconstructed breast, at the same axial level of the primary tumour

Figure 4 shows the MRI at time of diagnosis and local recurrence, showing the recurrence at the same plane as the primary tumour.

The patient underwent “lumpectomy” for the recurrence with implant preservation, followed by postoperative RT to the reconstructed breast, with a boost to the tumour bed.

## Discussion

In this *BRCA* PV breast cancer cohort, the evaluated residual breast volume after SSM/NSM was high and was *positively* associated with high BMI and risk reduction mastectomy, and *negatively* associated with the number of removed positive nodes. No differences were found between left side and right side in the therapeutic mastectomies. None of the patients had a positive margin reported after surgery. High residual breast volume was *not* found to be associated with a higher rate of locoregional relapse or any breast cancer event. Locoregional events occurred early after primary surgery, and most local recurrence occurred in proximity to the primary tumour bed, and no locoregional relapses occurred when postoperative RT was given. Suggesting that the early recurrences emerged from residual cancer cells left behind at primary surgery.

Breast MRI is considered the leading imaging-based assessment for breast tissue, but it may overestimate the residual glandular tissue as it often includes the subcutaneous tissue as part of the estimation [8, 11]. The breasts are not equal in size in many females, (left breasts tend to be larger) and the distribution of the glandular tissue (i.e. glandular density) varies within the breast. A high breast glandular density was described to be most often in the upper outer quadrant [35]. In our series, we did not find a significant difference in the residual volume for the left versus the right breast. The highest volume within a specific location/breast quadrant was not evaluated.

Our estimated residual breast volume after SSM/NSM was relatively high compared to reports of an intact breast volume on MRI (intact breast volume reported in a range of 112 to 2127 ml [5]). However, our measurement included the subcutaneous fat which also explains the association with BMI. The residual breast volume in our study is within the range of residual breast volume after SSM/NSM reported by Dietzel et al. [5], who found a mean residual breast volume of 427 ml and a maximum volume of 1078 ml after SSM/NSM. The volume of the true residual glandular component of the breast tissue is probably much lower than the “residual breast volume” [5]. However, the AI system is intentionally trained to include the subcutis. This was mainly due to two issues: (1) the histopathology assessment by Tramm et al. [25], showing that the limits of anatomic extension of the fibroglandular tissue may be imprecise and breast glands may intertwine with skin adnexa, adding that the thickness of the subcutis is variable and glandular tissue may be found in various amounts within the subcutis. (2) The abilities of commercially available breast MRI sequences cannot provide accurate volumes of glandular breast tissue only. Future developments in MRI, such as metabolic MRI and MR spectroscopic, might allow for a better estimation by more precisely discrimination glandular from fat tissue [36].

The fibroglandular tissue extending into the subcutis and skin adnexa is of significance for local recurrence. Tramm et al. [25] showed how DCIS, an in situ neoplasia, seemed like it was “invading” the skin due to involvement of the breast glands embedded within the subcutis and reaching up to the dermis. Moreover, a study evaluating the rate of residual *tumour cells* in the skin and the subcutaneous tissue (10 mm thickness) of total mastectomy specimens without dermal involvement reported that 20% of the cases had tumour cells within 10 mm below the skin [37]. These residual *tumour cells* were mostly found in the subcutis superficial to the primary tumour [37–39]. This might explain the location of the local recurrences observed in our study. Even though all cases reported to have “negative margins”, the pathology report did not include the superficial margins of the mastectomy specimen, and a negative “margin” does not exclude the possibility of residual *tumour cells* in the skin flap, which raises the concern that a thick flap has a higher chance of containing additional tumour foci [31, 37–39]. As patients who were treated with postmastectomy RT did not experience any local recurrences after SSM/NSM at the study follow-up, it is highly possible that the RT applied was sufficient to eradicate any residual tumour foci.

Our findings of a thicker flap in the risk-reducing mastectomy side, and a thinner flap associated with the number of involved nodes, are suggestive of the existence of a surgeon’s bias of radicality according to breast cancer stage. Our study did not find a correlation between the residual volume and breast cancer outcomes, including local recurrences. However, we should be cautious about concluding that the residual volume does not influence breast cancer outcomes, as one of the caveats of our study is the small sample size, overall similar volumes, and a low absolute number of breast cancer events.

Nevertheless, the finding of 5.6% local recurrences, mostly occurring early after primary surgery is of great concern, as the rates of local recurrences after mastectomy reduced over time due to better management and are generally estimated to be 3% at 10 years [40, 41].

In case of risk-reducing mastectomy, there are no guidelines if follow-up should include imaging as part of surveillance, if the flap thickness should be assessed or what should be the management in case of a thick flap [34, 42]. In our country, there are differences in screening and follow-up protocols among BRCA high-risk clinics [43]. Overall, the rate of breast cancer occurrence after risk reducing surgery is reported to be low, ranging from 0 to 5% [44–46]. It is unknown if a thicker flap poses a higher risk for breast cancer in this population [7, 34]. In our cohort, none of the patients experienced breast cancer in the risk-reducing side, but this might be due to the limited number of patients, competing events, protective effects of the oncological treatments for the primary cancer site (such as ovarian suppression), and a limited follow-up time.

This pilot study has several limitations, as it is retrospective with a limited number of patients and events, and the distribution of the residual breast volume was similar for the whole population (limited number of surgeons). We did not evaluate the differences between SSM and NSM (assuming NSM bare more residual tissue), pre/post-pectoral position, surgical incision site, surgeons’ expertise, nor if the differences between risk reducing and therapeutic mastectomy were due to different surgeons. The effect of tumour-related factors on local recurrences such as focality, distance from skin, or the presence of lymphovascular invasion was not done. As a tertiary centre that provides RT, oncogenetics, and medical oncology services, not all surgeries were performed at our hospital; therefore, not all information was available for the analysis.

While the generalizability of the results to other centres is uncertain, as much is dependent on surgical expertise and patient selection [2], this study is reporting a high residual breast volume after SSM/NSM, and a high local recurrence rate after SSM/NSM, occurring early after surgery. Even though no correlation found between an increase in residual volume and local recurrence event, this possibility could not be excluded.

The RT group did not have any local recurrences; however, RT should not be a salvage solution for non-radical surgery (radicality in terms of removing all tumour foci). Radiation therapy in case of implant-based reconstruction may lead to significant complications and implant loss [47]. Breast cancer surgery mandates expertise [2], and surgical approach should be pre-planned to achieve negative margins, including superficial margins. Radiation therapy should be offered only in cases where it is truly indicated after mastectomy [48, 49].

The BRILLIANT study is ongoing, and the system is planned to be able to assess the volume of the native breast prior the surgery, residual breast volume, and a tumour foci. This work complimentary to the SECRET study (Spatial location of breast cancer local rECurRence aftEr mastectomy, NCT06130111) is done in collaboration with the National Cancer Registry in the Netherlands (https://iknl.nl/en/ncr). The BRILLIANT AI system is aimed to assist in diagnosis, follow-up, and RT planning. We call for a multidisciplinary effort to improve the outcomes of locoregional therapies for breast cancer.

## Data Availability

Data sharing is not possible as this system is still under development for additional features and in IP submission process.
